# Endometrial Elasticity is an Ultrasound Marker for Predicting Clinical Pregnancy Outcomes after Embryo Transfer

**DOI:** 10.1007/s43032-024-01565-0

**Published:** 2024-05-20

**Authors:** Lin-lin Zhang, Shuo Huang, Li-ying Wang, Yuan-yuan Wang, Shan Lu, Rong Li

**Affiliations:** 1 Center for Reproductive Medicine, Department of Obstetrics and Gynecology, Peking University Third Hospital, Beijing, China; 2 Department of Obstetrics and Gynecology, Center for Reproductive Medicine, Peking University Third Hospital, North Garden Rd.49. Haidian District, 100191 Beijing, China

**Keywords:** Endometrial elasticity, Clinical pregnancy outcomes, Embryo transfer, Shear wave elasticity imaging

## Abstract

Endometrial elasticity is a potential new marker for assessing endometrial receptivity and pregnancy outcomes based on endometrial thickness and type. Currently, little research has been conducted on the elasticity of the endometrium using shear wave elasticity imaging (SWEI). This study aimed to explore whether endometrial elasticity is an ultrasound marker for predicting clinical pregnancy outcomes after embryo transfer. A total of 245 infertile women underwent ultrasonography before embryo transfer at the Peking University Third Hospital. We compared the endometrial elasticity and sub-endometrial blood flow rate using SWEI in the groups with different pregnancy outcomes. Trends in clinical pregnancy outcomes across the quartiles of endometrial elasticity in the fundus of the uterus (E1) were assessed. Logistic regression analysis was performed to obtain odds ratios for clinical pregnancy outcomes based on the quartiles of E1, with or without adjusting for potential confounding variables. Women in the clinical pregnancy group had higher E1 values and sub-endometrial blood flow rates in the uterine fundus than those in the non-pregnancy group. Women in the highest quartile of E1 had the most favorable clinical pregnancy rates. Endometrial elasticity measured using SWEI is a promising ultrasound marker for predicting clinical pregnancy outcomes after embryo transfer.

**Supplementary Information:**

The online version contains supplementary material available at 10.1007/s43032-024-01565-0.

## Introduction

Infertility is a reproductive health problem estimated to affect 186 million people around the globe [1]. In vitro fertilization-embryo transfer (IVF-ET) is one of the main technical methods for treating infertility. Despite significant progress in IVF-ET, implantation failure still affects numerous infertile couples [2, 3].

Embryo-related factors were reported to be crucial for implantation failure. Poor quality and chromosomal abnormalities of the embryo are well known to cause early implantation failures and miscarriages [4, 5]. Endometrium-related factors are also important. Endometrial receptivity is the ability of the endometrium to allow embryo implantation. Optimal receptivity can lead to a normal implantation process, which serves as the foundation for a healthy pregnancy [6]. Improvements in laboratory technology allow us to select better-quality embryos for transfer. Therefore, a convenient and non-invasive assessment of endometrial receptivity is critical for successful embryo implantation.

The complex adaptive changes and continuous evolution of the endometrium make it difficult to obtain useful information on receptiveness in real-time and in a personalized setting. Therefore, despite numerous studies exploring clinical methods to evaluate endometrial receptivity [7–10], little progress has been made, and the clinical evaluation of endometrial receptivity remains unclear and conflicting.

Ultrasound assessment of endometrial receptivity is widely used because of its simplicity, non-invasiveness, and repeatability [11–16]. It includes endometrial thickness, endometrial echo pattern, sub-endometrial blood flow pattern, endometrial volume, and uterine contractility [11, 17]. However, the poor abilities of these variables to predict clinical pregnancy outcomes prevent them from being used as diagnostic tests of endometrial receptivity [18].

Elasticity is an important characteristic of biological tissues. When pathological or physiological changes occur in the microstructure, such as the composition of tissue cells, macroscopically, the hardness of the tissue also changes accordingly [19]. By encoding different types of information and measuring the corresponding parameters, tissue elasticity can be qualitatively and quantitatively analyzed [20]. Currently, ultrasound elastography can be classified into strain elastography imaging and shear wave elastography imaging (SWEI).

Elastic ultrasound has been continuously applied in gynecological clinic, which can distinguish the pathophysiological changes of the endometrium and is important in evaluating the physiological state changes of tissues. It performs well in the differential diagnosis of uterine diseases, such as endometrial hyperplasia, endometrial carcinoma, submucosal leiomyoma, and endometrial polys [21, 22]. It is also helpful for determining the depth of the infiltrating myometrium in endometrial cancer.

Several studies have found that higher accuracy can be achieved by integrating endometrial elasticity and other clinical indicators to predict clinical pregnancy outcomes after embryo transfer [23, 24]. A study of clinical pregnancy outcomes in patients with frozen thawed embryo transfer (FET) combined with machine learning algorithms found that the accuracy rate of a logistic regression model predicting pregnancy outcome was 76.92% based on nine indicators including elastic grade and elastic ratio cut off value [23]. Shui X et al. developed and validated a pregnancy prediction model with a sensitivity of 83% and a specificity of 96% for predicting pregnancy by using age and ultrasonographic factors including ultrasound elastographic features [24]. Endometrial elasticity may be a potential novel marker for assessing endometrial receptivity and pregnancy outcomes. However, the effect of endometrial elasticity on the outcomes of IVF-ET was unclear due to very limited studies. Our study aimed to explore the correlation between endometrial elasticity and clinical pregnancy outcomes by applying SWEI, a non-invasive, repeatable, multimodal ultrasound method.

## Materials and Methods

### Ethical Approval

This study was approved by the Ethics Committee of the Peking University Third Hospital (2018S2-002). All clinical data were collected after obtaining informed consent.

### Study Population and Data Collection

**Fig. 1:**
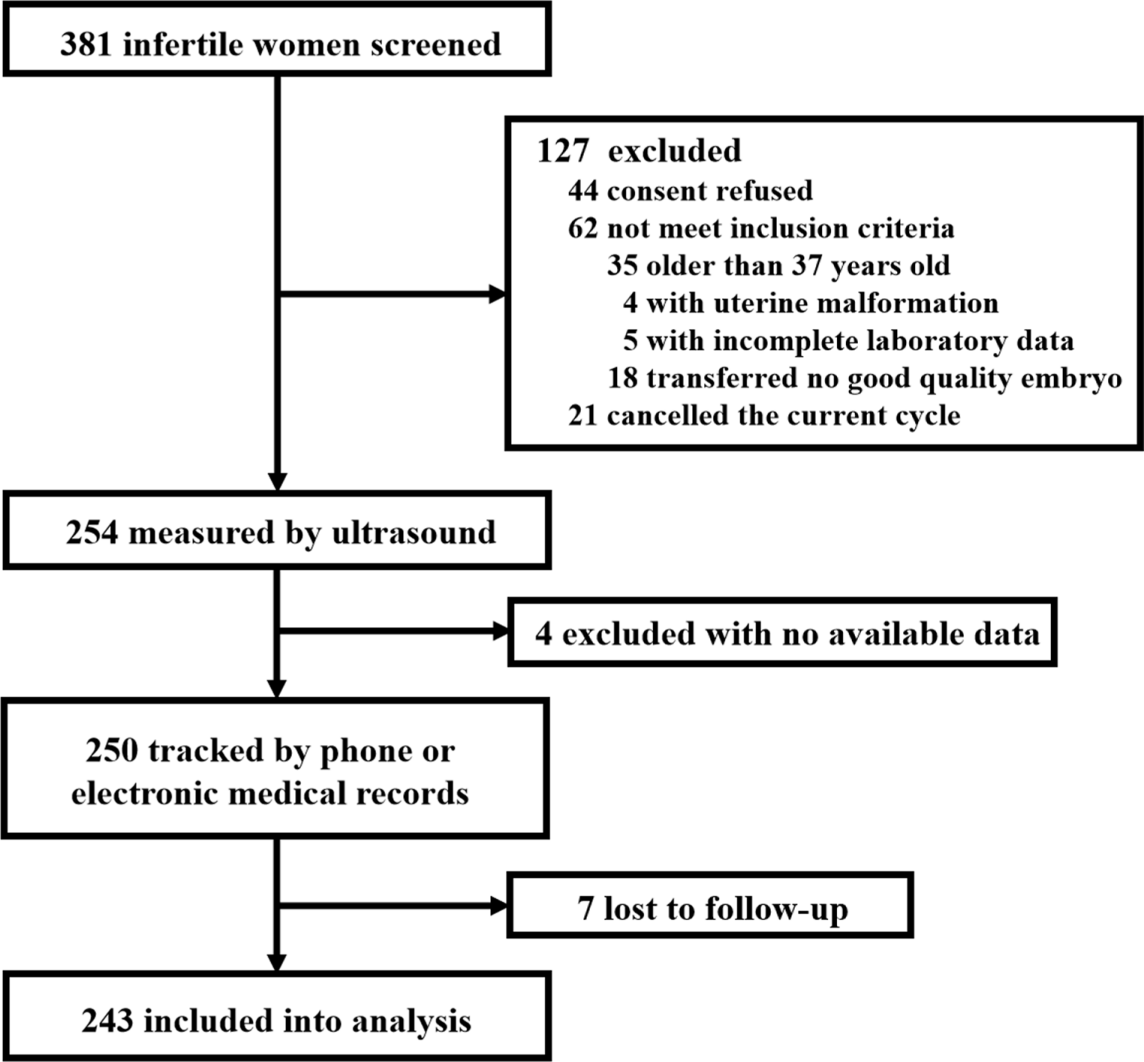
Flowchart summarizing study inclusion

We included 245 infertile women who underwent IVF-ET or FET from January 20 to 31, 2022, at Peking University Third Hospital (flowchart shown in Fig. 1). All subjects met the following inclusion criteria: (1) women aged 20–37 years; (2) failure to achieve pregnancy after 12 months or more of regular unprotected sexual intercourse [25]; and (3) women who were transferred with one or two good-quality embryos. The exclusion criteria were uterine malformations, abnormal uterine bleeding, malignant uterine masses, borderline or malignant adnexal cysts, loss to follow-up, and incomplete laboratory data.

Controlled superovulation protocols, trigger time and dosage, embryo culture, and transfer procedures were performed according to the standard protocols of the Reproductive Center of Peking University Third Hospital. When two and more follicles reached a diameter of at least 18 mm, 250 µg of recombinant human chorionic gonadotropin (rHCG, Ovidrel, Merck Serono, UK) was administered. Egg retrieval was performed 36 h after triggering, and the decision to apply for IVF or intracytoplasmic sperm injection was made based on the results of the semen analysis.

Typically, two embryos (day 3) were transferred to a fresh ET cycle. In the FET cycle, one blastocyst or two embryos were transferred after the endometrium had been prepared using a natural cycle, a hormone replacement cycle, or an ovulation stimulation cycle. In clinical practice, doctors also transferred one embryo depending on the patient’s condition (e.g., scarred uterus) or the patient’s request.

Blastocysts were graded according to three separate quality scores: developmental stage status of the blastocyst (1–6), grade of inner cell mass (A, B, C), and grade of trophectoderm (A, B, C) [26, 27]. The detailed scoring system is presented in Table S1 of Online Resource 1. Blastocysts with a stage status > 3, inner cell mass grade A or B, and trophectoderm grade A or B (≥ 4BB) were considered as good quality. The grading of the day 3 embryo quality was based on morphological parameters (blastomere number and fragments) [28, 29]. We defined the cleavage stage embryo as good quality if the embryo had six or more cells on day 3 and contained < 10% anucleated fragments.

In fresh ET cycles, the luteal support protocol was progesterone vaginal gel (Crinone 8%; Merck Serono), 90 mg per day. For the natural and stimulation cycles of FET, the luteal support regimen was 10 mg of oral dextrogestrel (Duphaston, Abbott Biologicals B. V.), twice daily. In hormone replacement cycles with FET, 90 mg progesterone vaginal gel, 20 mg oral dextrogestrel, and 6 mg estradiol valerate (Progynova) were administered per day.

Clinical data, including age, body mass index (BMI), gravidity, parity, types and causes of infertility, treatment modalities, antral follicle counts, basal hormone levels, and types and numbers of transferred embryos were collected.

### Ultrasonographic Data

**Fig. 2:**
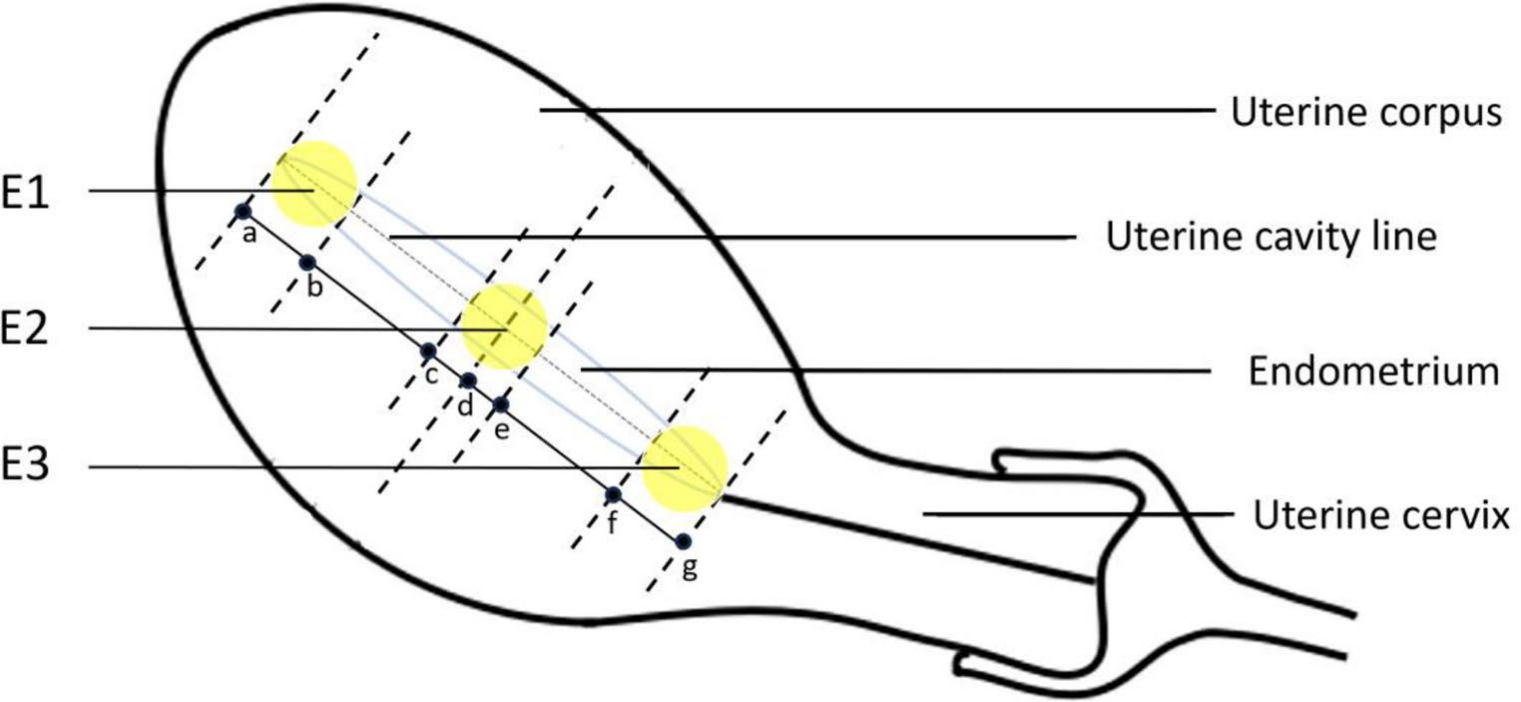
Sites of endometrium for the measurement of endometrial elasticity and sub-endometrial blood flow. *Notes* Line ag and uterine cavity line are parallel and have the same length; the seven dotted lines are perpendicular to line ag and endometrial line; point d is the middle point between point a and point g; point c localizes 0.5 cm away from the point d (close to a); point e localizes 0.5 cm away from the point d (close to g); point b localizes 1.0 cm away from the point a (close to d); point f localizes 1.0 cm away from the point g (close to d). The highlighted areas with a diameter of 1.0 cm are the three ROIs. The three dotted lines passing through point a, point c, and point f are the upper boundaries of the three ROIs, respectively. The three dotted lines passing through point b, point e, and point g are the lower boundaries of the three ROIs. E1, endometrial elasticity in the fundus of the uterus; E2, endometrial elasticity in the corpus of the uterus; E3, endometrial elasticity in the cervix of the uterus

We applied Voluson™ E10 (GE, Boston, MA, USA) to perform a color doppler ultrasound diagnosis. Endometrial thickness, sub-endometrial blood flow rate, and SWEI of endometrial elasticity were measured on the trigger day in a fresh ET cycle and on the day of ovulation or progesterone conversion in a FET cycle. All patients were examined according to the standard transvaginal examination performed by a single experienced sonographer. Endometrial thickness was noted in the midsagittal plane, and after the best possible resolution was ensured, the elastographic mode was switched. The default SWEI settings for the system were used, with the elasticity measurement range set between 0 and 180 kPa. The transducer was held gently to avoid external compression during the acquisition. To assist in anatomical localization, elastograms or color maps were displayed as an overlay in dual-mode alongside B-mode images in real time. Tissue elasticity can be assessed qualitatively and quantitatively using real-time color-coded maps and elasticity indices. On the SWEI color map, the elastic modulus is displayed in kilopascals using a default color scale with a graduation from blue to red, indicating low to high shear modulus (stiffness). SWEI was performed in the longitudinal view, and a region of interest (ROI) with a diameter of 1 cm was positioned. During the acquisition of the elastographic images, the patients were asked to hold their breath for approximately 5 s, and the propagation velocity of the shear elastic waves in the ROI was measured. After image stabilization, a color image of elasticity was obtained that was homogeneous and free of artifacts. As shown in Fig. 2, the first ROI was placed on the area between the muscle layer of the uterine fundus and the endometrium as the upper boundary, denoted as a region in the fundus of the uterus. The second ROI was located in the internal cervical orifice at the lower boundary, denoted as a region in the cervix of the uterus. A third ROI was placed between the first two ROIs, denoted as a region in the corpus of the uterus. Two measurements were performed in the same position with an examination interval > 3 s; the average was considered the corresponding regional endometrial elasticity value.

### Outcome Definitions

All participants were followed up for 3 months after transfer by a trained nurse through phone calls. Clinical pregnancy was defined as transvaginal ultrasound confirmation of an intrauterine gestational sac 4–5 weeks after ET [30]. We divided the women into clinical pregnancy and non-pregnancy groups. The primary outcomes were endometrial elasticity (E) and sub-endometrial blood flow rate (V) in the two groups. Two parameters were assessed for the endometrium in the uterine fundus, corpus uteri, and cervix (E1 and V1, endometrial elasticity and sub-endometrial blood flow in the fundus of the uterus; E2 and V2, endometrial elasticity and sub-endometrial blood flow in the corpus of the uterus; and E3 and V3, endometrial elasticity and sub-endometrial blood flow in the cervix of the uterus).

### Statistical Analysis

All statistical analyses were performed using SPSS Statistics version 24 (IBM Corp., Armonk, NY, USA). All continuous variables were analyzed using the Mann–Whitney U test and are shown as the median (25th percentile ∼ 75th percentile). The frequency distribution, constituent ratios, and percentages were used for categorical variable/binary data. The chi-square test was used to analyze count data. Statistical significance was set at *P* < 0.05.

We divided the distribution of E1 values into four groups from the lowest quartile (quartile 1, Q1) to the highest quartile (quartile 4, Q4). Participants’ data were analyzed according to quartile groups. Logistic regression analysis was performed to obtain the odds ratios (ORs) for clinical pregnancy outcomes based on E1 quartiles, with or without adjusting for potential confounding variables. The following adjustments were made: (1) no variables were adjusted; and (2) endometrial thickness, antral follicle numbers, basal hormone level, and BMI were adjusted. All ORs are reported with 95% confidence intervals (CI).

## Results

### Clinical Characteristics

Participants with intrauterine pregnancies on ultrasonography comprised the clinical pregnancy group. Participants with positive serum HCG results but no intrauterine pregnancy on ultrasonography and those with negative serum HCG results comprised the non-pregnancy group. There were no significant differences in baseline clinical characteristics between the two groups (*P* > 0.05), as shown in Table 1.

**Table 1:** Baseline clinical characteristics of infertile women with different clinical pregnancy outcomes

Baseline data	Clinical pregnancy *N* = 122	Non-pregnancy *N* = 121	Z	*P* value
Age (years)	32 (30 ∼ 34)	32 (30 ∼ 34)	−0.026	0.979
BMI (kg/m^2^)	22.3 (20.2 ∼ 24.5)	22.05 (20.3 ∼ 25.3)	−0.488	0.626
Gravidity				0.375
0	80 (65.6%)	77 (63.6%)		
1	29 (23.8%)	27 (22.3%)		
≥ 2	13 (10.6%)	17 (14.1%)		
Parity				0.584
0	115 (94.3%)	114 (94.2%)		
1	6 (4.9%)	7 (5.8%)		
≥ 2	1 (0.8%)	0 (0.0%)		
Infertility type				0.644
Primary infertility	81 (66.4%)	80 (66.1%)		
Secondary infertility	41 (33.6%)	41 (33.9%)		
Infertility period (years)	3 (2 ∼ 5)	3 (2 ∼ 5)	−0.772	0.440
Cause of infertility				0.424
Fallopian tubal infertility	11 (9.0%)	14 (11.6%)		
Uterine factors	0 (0.0%)	3 (2.5%)		
Ovulatory dysfunction and diminished ovarian reserve	10 (8.2%)	6 (4.9%)		
Male factor	35 (28.7%)	27 (22.3%)		
Unexplained factor	11 (9.0%)	12 (9.9%)		
Multiple factors	55 (45.1%)	59 (48.8%)		
Cycle type				0.146
Fresh IVF-ET (%)	52 (42.6%)	41 (33.9%)		
FET (%)	70 (57.3%)	80 (66.1%)		
Treatment modalities				0.509
Fresh IVF-ET				
Regular stimulation cycle	48 (39.3%)	39 (36.1%)		
Mild stimulation cycle	4 (3.3%)	2 (1.8%)		
FET				
Hormone replacement cycle	34 (27.9%)	39 (36.1%)		
Natural cycle	28 (23.0%)	22 (20.4%)		
Ovarian stimulation cycle	8 (6.5%)	6 (5.6%)		
Anti-mullerian hormone (ng/mL)	2.84 (1.91 ∼ 4.35)	3.5 (1.73 ∼ 5.25)	−0.990	0.322
Basal follicle stimulating hormone (FSH) (IU/L)	5.4 (4.2 ∼ 6.5)	5.45 (4.02 ∼ 6.76)	−0.254	0.800
Basal estradiol (E2) (nmol/L)	162 (118 ∼ 522)	171 (113 ∼ 252)	−0.703	0.482
Antral follicle count	12 (8 ∼ 17)	12 (8 ∼ 16)	−0.662	0.508
Numbers of Embryo to transfer				0.591
Single (%)	41 (33.9%)	47 (38.8%)		
Double (%)	81 (66.1%)	74 (61.2%)		
Type of Embryo to transfer				0.88
D3 embryo (%)	89 (73.0%)	90 (74.4%)		
Blastocyst (%)	33 (27.0%)	31 (25.6%)		

N is the total number of qualified subjects for evaluation

All continuous variables are reported as median (interquartile range)

All categorical variables are reported as numbers (percentages)

### Ultrasound Data for Different Clinical Pregnancy Outcomes

Endometrial thickness, E, and V were evaluated using SWEI. For each measurement, the mean E and V in the selected region were obtained from frozen images. The E1 levels were higher in women with clinical pregnancy than in those without pregnancy (24.92 vs. 19.38, *P* = 0.044). V1 was higher in the clinical pregnancy group than in the non-pregnancy group (2.88 vs. 2.54, *P* = 0.037) (Table 2).

**Table 2:** Ultrasound data of infertile women with different clinical pregnancy outcomes

	Clinical pregnancy *N* = 122	Non-pregnancy *N* = 121	Z	*P* value
Endometrial thickness (cm)	1.3 (1.1 ∼ 1.4)	1.30 (1.1 ∼ 1.4)	−0.189	0.850
E_1_	24.92 (13.35 ∼ 43.41)	19.38 (11.715 ∼ 33.42)	−2.013	0.044*
V_1_	2.88 (2.105 ∼ 3.800)	2.54 (1.975 ∼ 3.295)	−2.090	0.037*
E_2_	9.15 (5.35 ∼ 16.22)	8.85 (6.225 ∼ 14.25)	−0.326	0.744
V_2_	1.74 (1.335 ∼ 2.33)	1.72 (1.44 ∼ 2.18)	−0.290	0.772
E_3_	27.12 (15.05 ∼ 49.49)	26.2 (14.43 ∼ 53.125)	−0.020	0.984
V_3_	3.01 (2.24 ∼ 4.06)	2.96 (2.195 ∼ 4.21)	−0.016	0.987
E_1 + 2_	23.7 (16.67 ∼ 38.60)	22.1 (15.97 ∼ 31.58)	−0.879	0.379
V_1 + 2_	2.355 (1.94 ∼ 3.00)	2.235 (1.815 ∼ 2.79)	−1.696	0.090
E_mean_	21.18 (18.62 ∼ 31.10)	15.78 (10.645 ∼ 25.98)	−1.860	0.063

E_1_ and V_1_, endometrial elasticity and sub-endometrial blood flow in the uterine fundus

E_2_ and V_2_, endometrial elasticity, and sub-endometrial blood flow in the corpus of the uterus

E_3_ and V_3_, endometrial elasticity and sub-endometrial blood flow in the uterine cervix

E_1 + 2_, mean of E_1_ and E_2_; V_1 + 2_, mean of V_1_ and V_2_; E_mean_, mean of E_1_, E_2_, and E_3_

Data were analyzed using the Mann–Whitney U test and are shown as medians (interquartile ranges)

*P < 0.05

### Clinical Pregnancy Outcomes Based on Quartiles of Endometrial Elasticity in the Fundus of the Uterus (E1)

**Fig. 3:**
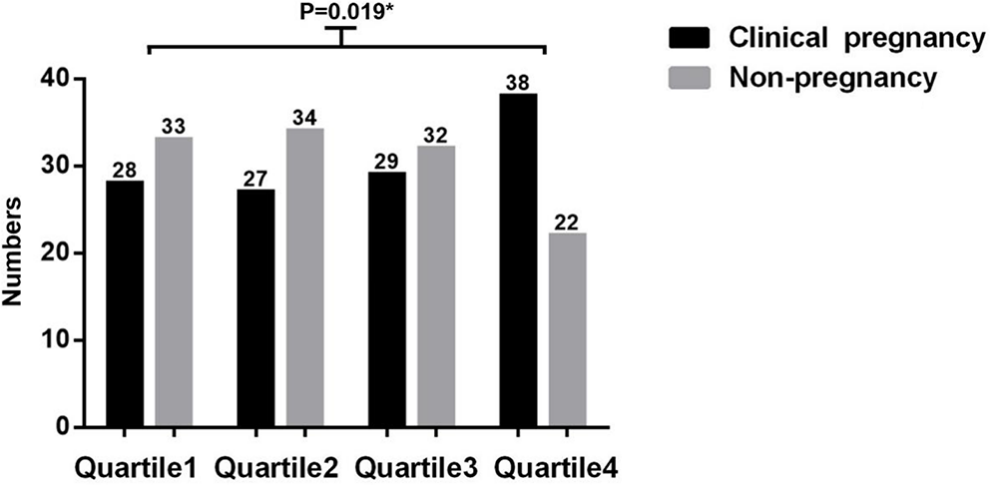
Number of infertile women with different clinical pregnancy outcomes based on quartiles of endometrial elasticity in the uterine fundus (E1)

Quartiles of endometrial elasticity level in the fundus of the uterus were as follows: Q1, ≤ 12.45 Kpa; Q2, 12.45–20.90 Kpa; Q3, 20.9–40.60 Kpa; and Q4, ≥ 40.61 Kpa. Figure 3 shows the number of infertile women with different pregnancy outcomes based on the E1 quartiles. There was a significant difference in the clinical pregnancy rate between Q4 and other quartiles (*P* = 0.019).

### Associations between Endometrial Elasticity and Clinical Pregnancy Outcomes

Table 3 shows the ORs for clinical pregnancy outcomes based on E1 quartiles. In the unadjusted model, compared to the women in Q4, the women in Q1 (OR, 0.435; 95% CI, 0.210–0.900), Q2 (OR, 0.407; 95% CI, 0.196–0.843), and Q3 (OR, 0.380; 95% CI, 0.183–0.790) had a significantly lower clinical pregnancy rate. According to previous studies and clinical experience, age, endometrial thickness, BMI, basal sex hormone levels, and antral follicle count can significantly affect pregnancy outcomes [18, 31–37]. Therefore, although there were no statistically significant differences in baseline characteristics in our study, an adjusted model was established. Similar results were obtained after adjusting for endometrial thickness, BMI, basal sex hormone levels, and antral follicle count. The women in Q1 (OR, 0.322; 95% CI, 0.135–0.768), Q2 (OR, 0.407; 95% CI, 0.196–0.843), and Q3 (OR, 0.380; 95% CI, 0.18–-0.790) had lower clinical pregnancy rates compared to the women in Q4.

**Table 3:** Model of the association between E1 and pregnancy outcomes analyzed by logistic regression

Parameter		*P* value	OR (95% CI)	Adjusted P value	aOR (95% CI)
*Pregnancy Outcome*					
Clinical pregnancy	Quartile 1	0.025*	0.435 (0.210–0.900)	0.011*	0.322 (0.135–0.768)
	Quartile 2	0.016*	0.407 (0.196–0.843)	0.006*	0.307 (0.132–0.713)
	Quartile 3	0.010*	0.380 (0.183–0.790)	0.004*	0.287 (0.124–0.665)
	Quartile 4	Reference		Reference	

*P < 0.05. aOR, adjusted OR

## Discussion

### Principal Findings

We found that endometrial elasticity and sub-endometrial blood flow in the fundus of the uterus were higher in the clinical pregnancy group than in the non-pregnancy group following ET. Furthermore, women in the highest quartile (Q4) of E1 had the best clinical pregnancy rates.

### Clinical and Research Implications

Uterine peristalsis was measured for 5 min by a specialized examiner before ET using transvaginal ultrasonography, and the results were recorded by experienced observes [38]. Previous studies have shown that the uterine peristaltic wave frequency before ET is inversely related to clinical pregnancy rates during ET [17, 38–42]. However, it is highly subjective and time consuming, and no consensus has been reached regarding its widespread clinical application. The endometrium itself cannot cause peristaltic movements because of the absence of smooth muscle cells. It is likely that the observed endometrial movements were caused by subtle contractions of the myofibrils. Endometrial wavelike activity reflects uterine peristalsis and the integrated receptivity of the uterus as a whole [43]. Therefore, endometrial elasticity is a more stable and easily detectable indicator of uterine peristalsis.

Consistent with our findings, Swierkowski-Blanchard et al. demonstrated that women with clinical pregnancy following intrauterine insemination were more likely to have low-frequency and high-intensity uterine contractions than those who failed to conceive [17]. Several potential mechanisms may account for endometrial elasticity in clinical pregnancies, which may be related to the facilitation of embryo transport. Endometrial elasticity may be affected by the elasticity of the myometrium according to its physiological structure. Higher endometrial elasticity can physically confine the pre-implanted embryo to the fundal region of the uterus, preventing it from being expelled, and allowing the embryo to finalize its implantation [44]. Endometrial and sub-endometrial blood perfusion are necessary for embryo implantation [45, 46], which explains the higher V1 in the clinical pregnancy group than in the non-pregnancy group. However, we also speculated that the higher elasticity contributing to embryo implantation was related to rich blood perfusion in the corresponding region. A large number of proliferating neonatal capillaries, uneven thicknesses, and disordered arrangements can cause increased interstitial pressure and enhanced hardness [22]. When a blastocyst is implanted into the uterus, endometrial stromal cells undergo extensive proliferation, differentiation, vascular remodeling, and immune cell recruitment in a process known as decidualization, which is essential for a successful pregnancy [47]. Mechanics are now widely recognized as important regulators of cell behavior, regulating both cell differentiation and migration [48]. A change in substrate stiffness can induce actin cytoskeletal reorganization and contractility, resulting in a change in the number of focal adhesion points and subsequently affecting migration speed and direction [47]. However, little is known about their effects on blastocyst implantation and trophoblast migration during placental development because of the lack of mechanical characterization of the human maternal–fetal interface. However, the relationship between endometrial elasticity, embryo implantation, and clinical pregnancy requires further investigation.

### Strengths and Limitations

Currently, ultrasound elastography can be classified into strain elastography imaging that uses internal or external compression stimulation, and SWEI that uses ultrasound-generated traveling shear wave stimulation. Strain elastography imaging quantifies the relative stiffness of ROIs by comparing the ratio of the strain index of the ROIs to the surrounding tissue, but does not provide an absolute value of stiffness. SWE, on the other hand, measures the shear wave speed and results in qualitative and quantitative estimates of tissue elasticity [49]. Smith et al. demonstrated no significant difference in the mean endometrial elasticity using SWEI among women in different age groups [50]. Yan et al. revealed that endometrial elasticity measured using SWEI increased with an increasing number of abortions, and there was no statistically significant difference in endometrial thickness, which may indicate a change in the endometrium shown by elasticity earlier than endometrial thickness [51]. To the best of our knowledge, this is the first study to investigate the effects of endometrial elasticity on embryo implantation in fresh and frozen-thawed ETs using SWEI. The ultrasound data were obtained by a single sonographer to ensure measurement stability. To avoid confounding factors such as age and embryo quality, we chose relatively young women (< 38 years) with at least one good-quality embryo. A multiple logistic regression analysis was performed to eliminate the effects of other confounding factors. Similar to our study, Li M et al. conducted a study to explore the correlation between endometrial elasticity and clinical pregnancy outcomes [23]. The difference is that their study measured endometrial elasticity by strain elastography imaging, and they measured elasticity characteristics in 78 women who underwent FET. Although they found that the accuracy of the clinical pregnancy outcome prediction model was higher with the inclusion of elasticity characteristics, there was no significant difference in elasticity-related indicators between the clinical pregnancy and non-pregnancy groups in their study. However, our study used SWE to measure endometrial elasticity on embryo implantation in fresh and frozen-thawed ETs and found statistically significant differences in E1 between the two groups, which provides an experimental basis for a subsequent study with a large sample size to explore cut off values of endometrial elasticity in the uterine fundus.

Our study had several limitations. It is important to note that the uterus is three-dimensional, and current techniques cannot simultaneously sample the endometrium at different sites. The ultrasound data were obtained by a single sonographer and the transducer was held gently to avoid external compression and ensure measurement stability. Nevertheless, we acknowledge that there is an operator-dependency of free-hand ultrasound systems in the measurement of endometrial elasticity. An additional limitation is that as a real-world study with a new clinical method, we included a limited sample size, making it difficult to conduct a subsequent subgroup analysis to further exclude confounding factors, although we only included young women with good-quality embryos. We plan to design a prospective study with a reasonable sample size in the FET of preimplantation genetic testing to exclude the impact of embryo chromosomal constitution.

## Conclusion

Women in the clinical pregnancy group had higher endometrial elasticity values in the uterine fundus. The measurement of endometrial elasticity by SWEI highlights its potential as a noninvasive ultrasound marker for predicting clinical pregnancy outcomes after ET.

## Electronic Supplementary Material

Below is the link to the electronic supplementary material.

Supplementary Material 1

## References

[1] Inhorn MC, Patrizio P. Infertility around the globe: new thinking on gender, reproductive technologies and global movements in the 21st century. Hum Reprod Update. 2015;21(4):411–26. 25801630 10.1093/humupd/dmv016

[2] Zegers-Hochschild F, Adamson GD, Dyer S, Racowsky C, de Mouzon J, Sokol R, et al. The International Glossary on Infertility and Fertility Care, 2017. Hum Reprod. 2017;32(9):1786–801. 29117321 10.1093/humrep/dex234 PMC5850297

[3] Coughlan C, Ledger W, Wang Q, Liu F, Demirol A, Gurgan T, et al. Recurrent implantation failure: definition and management. Reprod Biomed Online. 2014;28(1):14–38. 24269084 10.1016/j.rbmo.2013.08.011

[4] Das M, Holzer HE. Recurrent implantation failure: gamete and embryo factors. Fertil Steril. 2012;97(5):1021–7. 22425200 10.1016/j.fertnstert.2012.02.029

[5] Franasiak JM, Alecsandru D, Forman EJ, Gemmell LC, Goldberg JM, Llarena N, et al. A review of the pathophysiology of recurrent implantation failure. Fertil Steril. 2021;116(6):1436–48. 34674825 10.1016/j.fertnstert.2021.09.014

[6] Lessey BA, Young SL. What exactly is endometrial receptivity? Fertil Steril. 2019;111(4):611–7. 30929718 10.1016/j.fertnstert.2019.02.009

[7] Jin XY, Zhao LJ, Luo DH, Liu L, Dai YD, Hu XX, et al. Pinopode score around the time of implantation is predictive of successful implantation following frozen embryo transfer in hormone replacement cycles. Hum Reprod. 2017;32(12):2394–403. 29040606 10.1093/humrep/dex312

[8] Ruiz-Alonso M, Blesa D, Díaz-Gimeno P, Gómez E, Fernández-Sánchez M, Carranza F, et al. The endometrial receptivity array for diagnosis and personalized embryo transfer as a treatment for patients with repeated implantation failure. Fertil Steril. 2013;100(3):818–24. 23756099 10.1016/j.fertnstert.2013.05.004

[9] Wang XB, Qi QR, Wu KL, Xie QZ. Role of osteopontin in decidualization and pregnancy success. Reproduction. 2018;155(5):423–32. 29420252 10.1530/REP-17-0782

[10] Krylova Y, Polyakova V, Kvetnoy I, Kogan I, Dzhemlikhanova L, Niauri D, et al. Immunohistochemical criteria for endometrial receptivity in I/II stage endometriosis IVF-treated patients. Gynecol Endocrinol. 2016;32(sup2):33–6. 27759459 10.1080/09513590.2016.1232576

[11] Polanski LT, Baumgarten M. Endometrial receptivity testing and therapy in assisted Reproductive Treatment. Semin Reprod Med. 2021;39(1–02):27–33. 34391208 10.1055/s-0041-1730421

[12] Casper RF. Frozen embryo transfer: evidence-based markers for successful endometrial preparation. Fertil Steril. 2020;113(2):248–51. 32106971 10.1016/j.fertnstert.2019.12.008

[13] Hernández-Vargas P, Muñoz M, Domínguez F. Identifying biomarkers for predicting successful embryo implantation: applying single to multi-OMICs to improve reproductive outcomes. Hum Reprod Update. 2020;26(2):264–301. 32096829 10.1093/humupd/dmz042

[14] Hviid Saxtorph M, Persson G, Hallager T, Birch Petersen K, Eriksen JO, Larsen LG, et al. Are different markers of endometrial receptivity telling us different things about endometrial function? Am J Reprod Immunol. 2020;84(6):e13323. 33245608 10.1111/aji.13323

[15] Saare M, Lawarde A, Modhukur V, Mikeltadze I, Karro H, Minajeva A, et al. The expression pattern of endometrial receptivity genes is desynchronized between endometrium and matched endometriomas. Reprod Biomed Online. 2022;45(4):713–20. 35927210 10.1016/j.rbmo.2022.05.028

[16] Edgell TA, Rombauts LJ, Salamonsen LA. Assessing receptivity in the endometrium: the need for a rapid, non-invasive test. Reprod Biomed Online. 2013;27(5):486–96. 23933033 10.1016/j.rbmo.2013.05.014

[17] Swierkowski-Blanchard N, Boitrelle F, Alter L, Selva J, Quibel T, Torre A. Uterine contractility and elastography as prognostic factors for pregnancy after intrauterine insemination. Fertil Steril. 2017;107(4):961–e83. 28283264 10.1016/j.fertnstert.2017.02.002

[18] Craciunas L, Gallos I, Chu J, Bourne T, Quenby S, Brosens JJ, et al. Conventional and modern markers of endometrial receptivity: a systematic review and meta-analysis. Hum Reprod Update. 2019;25(2):202–23. 30624659 10.1093/humupd/dmy044

[19] Ophir J, Céspedes I, Ponnekanti H, Yazdi Y, Li X. Elastography: a quantitative method for imaging the elasticity of biological tissues. Ultrason Imaging. 1991;13(2):111–34. 1858217 10.1177/016173469101300201

[20] Taljanovic MS, Gimber LH, Becker GW, Latt LD, Klauser AS, Melville DM, et al. Shear-Wave Elastography: Basic Physics and Musculoskeletal Applications. Radiographics. 2017;37(3):855–70. 28493799 10.1148/rg.2017160116 PMC5452887

[21] Vora Z, Manchanda S, Sharma R, Das CJ, Hari S, Mathur S, et al. Transvaginal Shear Wave Elastography for Assessment of Endometrial and Subendometrial pathologies: a prospective pilot study. J Ultrasound Med. 2022;41(1):61–70. 33645765 10.1002/jum.15679

[22] Zhao HX, Du YY, Guo YJ, Zhou JH, Sun CQ, Wen XD, et al. Application value of Real-Time Shear Wave Elastography in diagnosing the depth of infiltrating muscular layer of Endometrial Cancer. J Ultrasound Med. 2021;40(9):1851–61. 33216384 10.1002/jum.15568

[23] Li M, Zhu X, Wang L, Fu H, Zhao W, Zhou C et al. Evaluation of endometrial receptivity by ultrasound elastography to predict pregnancy outcome is a non-invasive and worthwhile method. Biotechnol Genet Eng Rev. 2023:1–15. 10.1080/02648725.2023.2183585 36883689

[24] Shui X, Yu C, Li J, Jiao Y. Development and validation of a pregnancy prediction model based on ultrasonographic features related to endometrial receptivity. Am J Transl Res. 2021;13(6):6156–65. 34306354 PMC8290803

[25] Vander Borght M, Wyns C. Fertility and infertility: definition and epidemiology. Clin Biochem. 2018;62:2–10. 29555319 10.1016/j.clinbiochem.2018.03.012

[26] Gardner DK, Lane M, Stevens J, Schlenker T, Schoolcraft WB. Blastocyst score affects implantation and pregnancy outcome: towards a single blastocyst transfer. Fertil Steril. 2000;73(6):1155–8. 10856474 10.1016/s0015-0282(00)00518-5

[27] Gardner DK, Surrey E, Minjarez D, Leitz A, Stevens J, Schoolcraft WB. Single blastocyst transfer: a prospective randomized trial. Fertil Steril. 2004;81(3):551–5. 15037401 10.1016/j.fertnstert.2003.07.023

[28] Giorgetti C, Terriou P, Auquier P, Hans E, Spach JL, Salzmann J, et al. Embryo score to predict implantation after in-vitro fertilization: based on 957 single embryo transfers. Hum Reprod. 1995;10(9):2427–31. 8530679 10.1093/oxfordjournals.humrep.a136312

[29] Ziebe S, Petersen K, Lindenberg S, Andersen AG, Gabrielsen A, Andersen AN. Embryo morphology or cleavage stage: how to select the best embryos for transfer after in-vitro fertilization. Hum Reprod. 1997;12(7):1545–9. 9262293 10.1093/humrep/12.7.1545

[30] Devine K, Richter KS, Jahandideh S, Widra EA, McKeeby JL. Intramuscular progesterone optimizes live birth from programmed frozen embryo transfer: a randomized clinical trial. Fertil Steril. 2021;116(3):633–43. 33992421 10.1016/j.fertnstert.2021.04.013

[31] Ullah K, Rahman TU, Pan HT, Guo MX, Dong XY, Liu J, et al. Serum estradiol levels in controlled ovarian stimulation directly affect the endometrium. J Mol Endocrinol. 2017;59(2):105–19. 28539318 10.1530/JME-17-0036 PMC5510595

[32] Chan JM, Sukumar AI, Ramalingam M, Ranbir Singh SS, Abdullah MF. The impact of endometrial thickness (EMT) on the day of human chorionic gonadotropin (hCG) administration on pregnancy outcomes: a 5-year retrospective cohort analysis in Malaysia. Fertil Res Pract. 2018;4:5. 30116547 10.1186/s40738-018-0050-8 PMC6087003

[33] Vaegter KK, Lakic TG, Olovsson M, Berglund L, Brodin T, Holte J. Which factors are most predictive for live birth after in vitro fertilization and intracytoplasmic sperm injection (IVF/ICSI) treatments? Analysis of 100 prospectively recorded variables in 8,400 IVF/ICSI single-embryo transfers. Fertil Steril. 2017;107(3):641–e82. 28108009 10.1016/j.fertnstert.2016.12.005

[34] Zhou H, Zhang D, Luo Z, Yang A, Cui N, Hao G, et al. Association between Body Mass Index and Reproductive Outcome in women with polycystic ovary syndrome receiving IVF/ICSI-ET. Biomed Res Int. 2020;2020:6434080. 32908902 10.1155/2020/6434080 PMC7463361

[35] Chen H, Li J, Cai S, Zeng S, Yin C, Kuang W, et al. Impact of body mass index (BMI) on the success rate of fresh embryo transfer in women undergoing first in vitro fertilization/intracytoplasmic sperm injection (IVF/ICSI) treatment. Int J Obes (Lond). 2022;46(1):202–10. 34628467 10.1038/s41366-021-00978-0

[36] Li NJ, Yao QY, Yuan XQ, Huang Y, Li YF. Anti-müllerian hormone as a predictor for live birth among women undergoing IVF/ICSI in different age groups: an update of systematic review and meta-analysis. Arch Gynecol Obstet. 2023;308(1):43–61. 35907969 10.1007/s00404-022-06683-1

[37] Li HW, Lee VC, Lau EY, Yeung WS, Ho PC, Ng EH. Role of baseline antral follicle count and anti-mullerian hormone in the index stimulation cycle of IVF treatment in predicting outcome of subsequent frozen-thawed embryo transfers. Gynecol Endocrinol. 2014;30(7):490–3. 24641676 10.3109/09513590.2014.899572

[38] Zhu L, Che HS, Xiao L, Li YP. Uterine peristalsis before embryo transfer affects the chance of clinical pregnancy in fresh and frozen-thawed embryo transfer cycles. Hum Reprod. 2014;29(6):1238–43. 24664129 10.1093/humrep/deu058

[39] Chung CH, Wong AW, Chan CP, Saravelos SH, Kong GW, Cheung LP, et al. The changing pattern of uterine contractions before and after fresh embryo transfer and its relation to clinical outcome. Reprod Biomed Online. 2017;34(3):240–7. 28089077 10.1016/j.rbmo.2016.12.011

[40] MM IJ, Evers JL, Dunselman GA, Volovics L, Hoogland HJ. Relation between endometrial wavelike activity and fecundability in spontaneous cycles. Fertil Steril. 1997;67(3):492–6. 9091336 10.1016/s0015-0282(97)80075-1

[41] Kim A, Young Lee J, Il Ji Y, Hyeog Lee H, Sil Lee E, Yeol Kim H, et al. Do endometrial movements affect the achievement of pregnancy during Intrauterine Insemination? Int J Fertil Steril. 2015;8(4):399–408. 25780522 10.22074/ijfs.2015.4180 PMC4355927

[42] Fanchin R, Righini C, Olivennes F, Taylor S, de Ziegler D, Frydman R. Uterine contractions at the time of embryo transfer alter pregnancy rates after in-vitro fertilization. Hum Reprod. 1998;13(7):1968–74. 9740459 10.1093/humrep/13.7.1968

[43] Ijland MM, Evers JL, Dunselman GA, van Katwijk C, Lo CR, Hoogland HJ. Endometrial wavelike movements during the menstrual cycle. Fertil Steril. 1996;65(4):746–9. 8654632 10.1016/s0015-0282(16)58207-7

[44] MM IJ, Hoogland HJ, Dunselman GA, Lo CR, Evers JL. Endometrial wave direction switch and the outcome of in vitro fertilization. Fertil Steril. 1999;71(3):476–81. 10065785 10.1016/s0015-0282(98)00501-9

[45] Chen M, He Y, Zhang P, Geng Q, Liu Q, Kong L, et al. Comparison of Uterine receptivity between fertile and unexplained infertile women by Assessment of Endometrial and Subendometrial Perfusion using contrast-enhanced Ultrasound: which Index is better–peak intensity or area under the curve? Ultrasound Med Biol. 2016;42(3):654–63. 26723901 10.1016/j.ultrasmedbio.2015.11.008

[46] Cheng F, Li T, Wang QL, Zhou HL, Duan L, Cai X. Effects of hydrosalpinx on ultrasonographic parameters for endometrial receptivity during the window of implantation measured by power color doppler ultrasound. Int J Clin Exp Med. 2015;8(4):6103–8. 26131211 PMC4483946

[47] Gellersen B, Brosens IA, Brosens JJ. Decidualization of the human endometrium: mechanisms, functions, and clinical perspectives. Semin Reprod Med. 2007;25(6):445–53. 17960529 10.1055/s-2007-991042

[48] Wang X, Yu Q. An update on the progress of transcriptomic profiles of human endometrial receptivity. Biol Reprod. 2018;98(4):440–8. 29365037 10.1093/biolre/ioy018

[49] Sigrist RMS, Liau J, Kaffas AE, Chammas MC, Willmann JK. Ultrasound Elastography: review of techniques and clinical applications. Theranostics. 2017;7(5):1303–29. 28435467 10.7150/thno.18650 PMC5399595

[50] Manchanda S, Vora Z, Sharma R, Hari S, Das CJ, Kumar S, et al. Quantitative Sonoelastographic Assessment of the normal Uterus using Shear Wave Elastography: an initial experience. J Ultrasound Med. 2019;38(12):3183–9. 31077426 10.1002/jum.15019

[51] Jiao Y, Xue N, Zou C, Shui X, Wang H, Hu C. Assessment of early damage of endometrium after artificial abortion by shear wave elastography. Insights Imaging. 2020;11(1):28. 32128718 10.1186/s13244-020-0841-4 PMC7054526

