# Supplementary material for: Endometrial Elasticity is an Ultrasound Marker for Predicting Clinical Pregnancy Outcomes after Embryo Transfer
Source: Reprod Sci. 2024 May 20;32(1):64–73. doi: 10.1007/s43032-024-01565-0 (PMC11729198; doi:10.1007/s43032-024-01565-0)
Supplement: Supplementary file 1 — Supplementary Material 1 [file 43032_2024_1565_MOESM1_ESM.docx]

# *Reproductive Sciences*

# Endometrial elasticity is an ultrasound marker for predicting clinical pregnancy outcomes after embryo transfer

**Authors:** Lin-lin Zhang^1*^, Shuo Huang^1*^, Li-ying Wang^1^, Yuan-yuan Wang, Shan Lu^2^, Rong Li^1#^

**Affiliations:**

^1^ Center for Reproductive Medicine, Department of Obstetrics and Gynecology, Peking University Third Hospital, Beijing, China.

^2^ Department of Obstetrics and Gynecology, Peking University Third Hospital, Beijing 100191, China.

*Linlin Zhang and Shuo Huang contributed equally to this work;

**Corresponding author:**

#Corresponding to Rong Li:

Center for Reproductive Medicine, Department of Obstetrics and Gynecology, Peking University Third Hospital, Beijing, China.

North Garden Rd.49.

Haidian District, Beijing, 100191, China

[Tel: +86](Tel:+86) 010 156 1190 8999

E-mail: [roseli001@sina.com](mailto:roseli001@sina.com)

Table S1. Gardner score in detail.

| Expansion grade | Blastocyst development and stage status |
| --- | --- |
| 1 | Blastocoel cavity less than half the volume of the embryo |
| 2 | Blastocoel cavity more than half the volume of the embryo |
| 3 | Full blastocyst, cavity completely filling the embryo |
| 4 | Expanded blastocyst, cavity larger than the embryo, with thinning of the shell |
| 5 | Hatching out of the shell |
| 6 | Hatched out of the shell |
| Inner cell mass grade | Inner cell mass quality |
| A | Many cells, tightly packed |
| B | Several cells, loosely grouped |
| C | Very few cells |
| Trophectoderm grade | Trophectoderm quality |
| A | Many cells, forming a cohesive layer |
| B | Few cells, forming a loose epithelium |
| C | Very few large cells |

Reference:

Gardner DK, Lane M, Stevens J, Schlenker T, and Schoolcraft WB. Blastocyst score affects implantation and pregnancy outcome: towards a single blastocyst transfer. Fertil Steril 2000: 73; 1155-1158.
